# Ceftaroline-Associated Neutropenia: Case Series and Literature Review of Incidence, Risk Factors, and Outcomes

**DOI:** 10.1093/ofid/ofz168

**Published:** 2019-03-28

**Authors:** Eva L Sullivan, R Brigg Turner, Hollis R O’Neal, Nancy F Crum-Cianflone

**Affiliations:** 1 Pharmacy Department, Scripps Mercy Hospital, San Diego, California; 2 School of Pharmacy, Pacific University, Hillsboro, Oregon; 3 Pulmonary & Critical Care Medicine, Louisiana State University Health/Our Lady of the Lake Regional Medical Center, Baton Rouge, Louisiana; 4 Internal Medicine Department, Scripps Mercy Hospital, San Diego, California; 5 Infectious Disease Division, Scripps Mercy Hospital, San Diego, California

**Keywords:** ceftaroline, incidence, neutropenia, review, side effects

## Abstract

Ceftaroline is increasingly prescribed for “off-label” indications involving longer durations and higher doses. There have been postmarketing case reports of neutropenia among patients who have received extended durations of ceftaroline, but limited published data currently exist on its incidence and risk factors. We review a total of 37 published cases of ceftaroline-associated neutropenia including cases (n = 4) identified in our health care system. The median time from ceftaroline initiation to development of neutropenia (range) was 25 (8–125) days, with a median duration of neutropenia (range) of 4 (1–16) days. Agranulocytosis (absolute neutrophil count [ANC] nadir < 100 cells/mm^3^) developed in 49% of cases (n = 18), and there was an ANC nadir of 0 in 27% (n = 10). The overall incidence of neutropenia among cases receiving ceftaroline for ≥7–14 days (range) was 12% (7%–18% per individual study), higher than for comparator antibiotics in the literature. Risk factors for ceftaroline-associated neutropenia varied among studies and remain poorly defined.

The development of novel antibiotics is important in addressing the growing rates of antibiotic resistance. For instance, *Staphylococcus aureus* remains a leading cause of bacteremia and endocarditis, with an increasing preponderance due to methicillin-resistant *S. aureus* (MRSA) strains [1, 2]. Given the limitations of the currently available antibiotics (eg, vancomycin) for treating MRSA infections, including drug intolerance, adverse events, and/or clinical failure [3, 4], new antibiotics with anti-MRSA activity have been recently developed.

Ceftaroline (Teflaro^®^) gained Food and Drug Administration (FDA) approval in 2010 and is the first licensed cephalosporin that includes coverage against MRSA. Studies leading to its approval include 2 clinical trials on community-acquired bacterial pneumonia (CABP; FOCUS 1 and FOCUS 2) [5, 6] and 2 additional studies on acute bacterial skin and skin structure infections (ABSSSIs; CANVAS 1 and CANVAS 2) [7, 8]. These 4 studies evaluated a total of 1307 subjects, with the most common adverse events among those receiving ceftaroline being diarrhea, nausea, and rash; no patient developed neutropenia. All studies utilized a ceftaroline dosage of 600 mg intravenously (IV) every 12 hours for durations of 5–14 days [5–8]. More recently, approval for bacteremia in the setting of ABSSSI has been added as an indication for ceftaroline use [9].

Postmarketing data are important for detecting unexpected adverse events associated with newly marketed drugs, especially when they are utilized for “off-label” use, an increasingly common practice for multidrug-resistant organism (MDRO) infections with limited treatment options. As such, ceftaroline has been utilized to treat MRSA bacteremia, endocarditis, and orthopedic infections [3, 10]. For these indications, ceftaroline has often been utilized for longer treatment durations (up to ≥6 weeks). Further, higher doses of ceftaroline have often been prescribed (600 mg IV every 8 hours vs 12 hours). This higher dosing frequency is based on pharmacokinetic studies suggesting that every-8-hour dosing may optimize time-dependent killing in severe infections involving high bacterial inoculums [11, 12], although clinical data to support this approach are currently lacking.

Data on potential adverse events associated with ceftaroline use, especially when utilized for longer durations or at higher doses, are needed. Hematologic effects are of particular interest, as long durations of β-lactam antibiotics may cause leukopenia [13]. To date, postmarketing case reports of neutropenia among patients receiving extended durations of ceftaroline have been published [14, 15]; however, data remain limited.

Hence, we conducted a case series of patients in our health care system who received ceftaroline for ≥7 days and subsequently developed incident neutropenia. In addition, given the limited data on the incidence as well as the timing and risk factors of ceftaroline-associated neutropenia, we conducted a review of the published literature to provide a comprehensive summary of this emerging adverse event.

## METHODS

### Case Series: Study Setting and Patients

Our health care system is comprised of 4 large urban hospitals with a total of 1400 available beds located in Southern California. Ceftaroline is restricted to the infectious diseases service and is typically reserved for complicated MRSA infections that have failed or are intolerant to firstline therapies. Adult patients who received consecutive ceftaroline therapy for ≥7 days during January 2010 to December 2017 were screened for study inclusion. This 7-day cutoff was selected based on the phase III studies having a mean/median ceftaroline duration of approximately 7 days with no subjects developing neutropenia [5–8] and previous noncomparative retrospective studies [16–18] identifying a higher neutropenia risk in patients receiving >7 days of therapy. We excluded patients who had neutropenia immediately before ceftaroline initiation or in whom an alternate cause of neutropenia was likely based on assessment by an infectious diseases physician, along with subsequent retrospective review by 2 authors (E.S., N.C.). In addition, the Naranjo Adverse Drug Reaction Probability Scale was applied to identified cases; a score of 3 or 4 indicated that the drug was a possible cause of neutropenia, and a score of 5 to 7 indicated that the drug was a probable cause of neutropenia [19].

### Study Outcome

The primary outcome was the incidence of neutropenia during ceftaroline therapy. Neutropenia was defined as an absolute neutrophil count (ANC) of <1500 cells/mm^3^.

### Review of Literature

A search of the published English medical literature (PubMed, Embase, Ovid Medline) from 2010 (the year of ceftaroline approval) to December 2017 was conducted. Keywords included “ceftaroline” or “cephalosporin” and “neutropenia,” “leukopenia,” or “agranulocytosis.” References from identified papers were reviewed for additional publications.

To be included in this review, cases had to be adults (≥18 years) who received ≥7 days of ceftaroline and subsequently developed new-onset neutropenia (ANC < 1500 cells/mm^3^). The Naranjo Adverse Drug Reaction Probability Scale was applied as above [19]. Cases in duplicative publications (eg, Blumenthal et al. and Furtek et al.) were counted once [16, 20]. Cases with preexisting neutropenia before ceftaroline use or cases receiving other agents expected to cause neutropenia (eg, chemotherapy) were excluded, as reported within the respective papers. For publications without individualized data in the original published manuscript, authors were contacted electronically and individualized data were requested; cases in which no or very limited individualized data could be obtained were excluded from this review.

For each identified case, data collected included demographics, underlying medical conditions, weight/body mass index, creatinine clearance, microbiology and location of infection, and initial antibiotic therapy. Data collected regarding ceftaroline use included indication, duration and dosage, and concurrent antibiotics. In addition, we evaluated if the recommended dosage adjustments were made based on creatinine clearance (CrCl; ≤50 mL/min) [21]. Baseline (at ceftaroline initiation) and nadir counts for the white blood cell count (WBC), neutrophil percentage, ANC, hemoglobin, and platelets were collected. Symptoms and signs at the time of neutropenia (eg, fever, rash) and any complications from the neutropenia were collected. The use of alternate antibiotics (after ceftaroline discontinuation) and G-CSF (for the management of neutropenia) was noted. Finally, the outcome of the patient and infection was collected. Missing data were accounted for within the denominator for each variable when calculating percentages.

## RESULTS

### Case Series

A total of 61 patients received ceftaroline for ≥7 days in our health care system. Five of these patients were excluded: 4 patients for preexisting neutropenia and 1 patient who received a concurrent antimicrobial IV vancomycin, thought to be the cause of neutropenia based on evaluation by an infectious diseases physician.

Overall, 4 (7%) of the 56 patients developed incident neutropenia without another identifiable cause. The duration of ceftaroline use among these 4 cases was 8, 20, 21, and 23 days, respectively. The duration among those without neutropenia was a median (range) of 14 (7–103) days. The score on the Naranjo Adverse Drug Reaction Probability Scale was 7 for each case. Concurrent medications that may be associated with neutropenia were reviewed, and it is important to note that neutropenia resolved despite continuation of these agents. These cases are summarized below and in Table 1.

**Table 1.** Summary of Current Cases of Ceftaroline-Associated Neutropenia

Age Sex	Medical History	BMI, kg/m^2^	Ceftaroline Indication and Duration, d	Dose/CrCl, mL/min	Initial ANC and Nadir ANC, cells/mm^3^	Days of Neutropenia	G-CSF Use	Eosinophilia	Concurrent Drugs^a^/ Antibiotics^b^ at Time of Neutropenia	Antibiotics Post-ceftaroline	Outcome
66 M	Alcoholic/hepatitis C cirrhosis, polysubstance abuse, chronic kidney disease, diabetes mellitus	26.5	Prosthetic knee infection × 8 d	300 mg IV q8h/22	2976/1172	2	No	Yes (8.6%)	Quetiapine/none	Daptomycin	Favorable
59 F	Diabetes mellitus, drug use	28.1	Bacteremia, endocarditis, diskitis/osteomyelitis × 20 d	400 mg IV q8h/47	4284/20	10	Yes (2 doses)	Yes (36%)	None/daptomycin	Telavancin	Favorable
26 F	IV drug use	26.5	Bacteremia, endocarditis × 21 d	600 mg & 400 mg IV 8 h/100 & 45	17 484/5	7	Yes (7 doses)	No	Famotidine/daptomycin	Daptomycin + doxycycline	Favorable
44 M	Diabetes mellitus, alcoholic cirrhosis	25.1	Osteomyelitis, endocarditis, endophthalmitis, and septic arthritis × 23 d	600 mg IV q12 h/85	1886/1472	2	No	Yes (12%)	Famotidine/daptomycin	Daptomycin	Favorable

Abbreviations: ANC, absolute neutrophil count; BMI, body mass index; CrCl, creatinine clearance; G-CSF, granulocyte colony-stimulating factor; IV, intravenous.

^a^Drugs associated with neutropenia [33, 34].

^b^Antibiotics not associated with neutropenia.

#### Case 1

**Figure 1.**
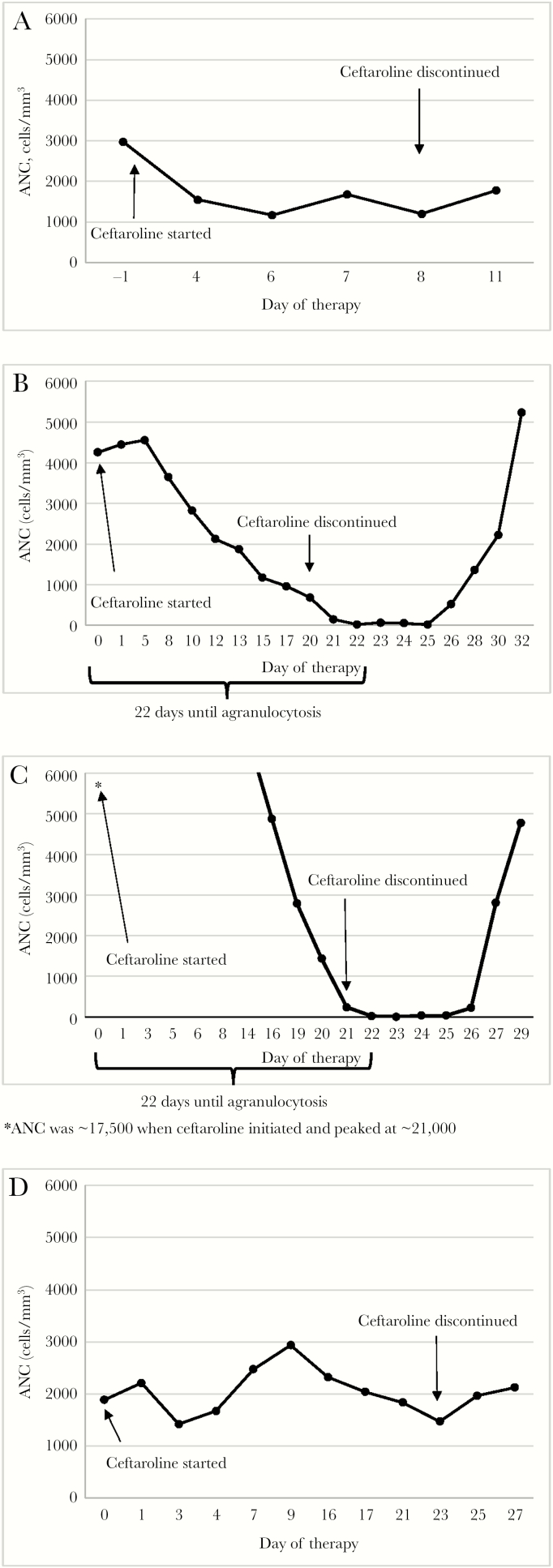
Absolute neutrophil count (ANC) trajectories for current cases.

A 66-year-old male was initiated on vancomycin IV for a MRSA prosthetic knee infection. Due to an increase in serum creatinine, vancomycin was switched to ceftaroline 300 mg IV every 8 hours (creatinine clearance of 22 mL/min) on hospital day 2. By day 9, the patient became neutropenic with an ANC of 1172 cells/mm^3^. On day 11, he continued to be neutropenic with an ANC of 1205 cells/mm^3^ (Figure 1A) and also developed eosinophilia (8.6%), at which time ceftaroline was switched to daptomycin. Follow-up WBC on day 14 was 2900 cells/mm^3^ (61.4% neutrophils).

#### Case 2

A 59-year-old female was treated with multiple antibiotic courses that included vancomycin and daptomycin for MRSA bacteremia with endocarditis. On day 24, antibiotics were changed to ceftaroline (renally adjusted at 400 mg IV every 12 hours × 3 days, then 400 mg IV every 8 hours for a CrCl of 47 mL/min) and daptomycin due to a newly discovered spinal infection including osteomyelitis and epidural abscess noted on imaging. On day 44 of hospitalization, she developed neutropenia with an ANC nadir of 20 cells/mm^3^, and ceftaroline was discontinued (Figure 1B). The patient’s neutropenia resolved after 10 days, with a follow-up ANC of 2223 cells/mm^3^, and the patient required broad-spectrum antibiotics and G-CSF due to neutropenic fevers. This case has been previously reported [22].

#### Case 3

A 26-year-old female was started on vancomycin IV for MRSA bacteremia complicated by endocarditis. On hospital day 3, ceftaroline 600 mg IV every 8 hours was added to vancomycin due to blood cultures remaining positive. Vancomycin was discontinued on day 14 because the patient developed acute renal failure. Daptomycin was then added to ceftaroline for additional coverage for MRSA endocarditis on day 20. After 21 days of ceftaroline therapy, the ANC had decreased to 4.5 cells/mm^3^, resulting in its discontinuation (Figure 1C). Neutropenia was present for 7 days, and G-CSF was administered daily for 7 days.

#### Case 4

A 44-year-old male with extensively disseminated MRSA infection including endocarditis, endophthalmitis, septic arthritis, and spinal osteomyelitis with abscesses was treated with vancomycin IV. On day 6, ceftaroline 600 mg IV every 8 hours was added due to persistent bacteremia. By day 8, vancomycin IV was changed to daptomycin due to continued positive blood cultures. Due to concern for eosinophilia (12%), ceftaroline was discontinued on day 54. On day 85, daptomycin was changed to combination therapy with linezolid and vancomycin IV due to worsening endophthalmitis. Vancomycin IV was subsequently changed to ceftaroline 600 mg IV every 12 hours on day 89. By day 90, the linezolid was changed to daptomycin due to cytopenias. Ceftaroline was discontinued at day 112 due to pancytopenia (WBC of 3100 cells/mm^3^, 48% neutrophils, ANC of 1472 cells/mm^3^) and eosinophilia (10%) (Figure 1D). By day 116, the pancytopenia and eosinophilia had improved, with a WBC at 4100 cells/mm^3^ with 52% neutrophils.

### Review of the Literature

In total, 37 cases were identified, including 5 single case reports [23–27]; 2 case series (3 and 4 cases, respectively) [14, 15] and 4 retrospective studies (with 4, 5, 7, and 9 cases, respectively) current study [16, 17, 28]. An additional 6 cases were found in the literature; however, individualized data could not be obtained [18, 29]. Detailed information for each case is provided in Supplementary Table 1.

Demographics among the 37 cases of ceftaroline-associated neutropenia included in this review are in shown in Table 2. All cases with reported information on medical history had an underlying condition; the types of conditions varied widely, with the most common being illicit drug use, reported in 5 cases.

**Table 2.** Summary Characteristics of Patients in the Systematic Review Receiving Ceftaroline who Developed Neutropenia, 2010–2017 (n = 37)

Demographics	
Median age (range), y	44 (20–90)
Sex, female, No. (%)	22 (59)
Infection characteristics, No. (%)	
Organism treated	
*S. aureus* (most commonly MRSA)	31 (83)
Streptococcus	1 (3)
Coagulase-negative *Staphylococcus*	1 (3)
Unknown	4 (11)
Type of infection,^a^ No. (%)	
Bacteremia	8 (22)
Heart/endocarditis	10 (27)
Orthopedic	18 (49)
Spinal/CNS	5 (14)
SSTI/wound	4 (11)
Endovascular NOS	5 (14)
Pneumonia	5 (14)
Ceftaroline dosage, No. (%)	
CrCl > 50 mL/min	
600 mg every 12 h	15/22 (68)
600 mg every 8 h	7/22 (32)
CrCl 31–50 mL/min	
600 mg every 12 h	1/3 (33)
400 mg every 8 h	2/3 (67)
CrCl 15–30 mL/min	
400 mg every 12 h	1/2 (50)
300 mg every 8 h	1/2 (50)
CrCl NR	
600 mg every 12 h	5/7 (71)
600 mg every 8 h	2/7 (29)
Dose NR	3/3 (100)
Neutropenia	
Median time to development (range), d	25 (8–125)
Nadir ANC < 100 cells/mm^3^, No. (%)	18 (49)
Mean duration of neutropenia (range), d	4 (1–16)
Concurrent antibiotic, yes, No. (%)	12/30 (40)
Concurrent renal insufficiency (CrCl < 60 mL/min), No. (%)	7/27 (26)
Concurrent hematologic effects, No. (%)	
New-onset anemia (drop of ≥2 mg/dL)	6/25 (24)
New-onset thrombocytopenia (drop of > 100 cells/ mm^3^)	8/26 (31)
Eosinophilia	9/26 (35)
Neutropenic complications, No. (%)	
Fever	6 (16)
Bacteremia	1 (3)
Receipt of G-CSF for neutropenia, No. (%)	11 (30)
Outcome, No. (%)	
Favorable	37 (100)

Denominator is n = 37, unless otherwise noted.

Abbreviations: ANC, absolute neutrophil count; CNS, central nervous system; CrCl, creatinine clearance; G-CSF, granulocyte colony-stimulating factor; MRSA, methicillin-resistant *Staphylococcus aureus*; NOS, not otherwise specified; NR, not reported.

^a^Some patients had multiple sites of infection.

The organism most frequently being treated with ceftaroline was *S. aureus*, most commonly MRSA. Only 10 cases provided information on the rationale for ceftaroline use: clinical failure of prior antibiotic therapy in 6 cases, renal failure or concern for worsening renal function in 2 cases, decreasing WBC and platelet counts in 1 case, and a minimal inhibitory concentration of 2 µg/mL for vancomycin in 1 case. Two (7%) of 27 patients received higher-than-FDA-approved recommended doses based on their creatinine clearance among those with available data.

The median time from ceftaroline initiation to the development of neutropenia (range) was 25 (8–125) days. Only 2 cases had a ceftaroline duration of ≤14 days, 6 had a duration of 15–20 days, and 28 cases had been receiving ceftaroline for ≥21 days (1 case did not report the duration). Of note, many of the reviewed studies did not include patients who received ceftaroline for less than 7 or 14 days. The median duration of neutropenia (range) was 4 (1–16) days. Agranulocytosis (ANC nadir < 100 cells/mm^3^) developed in 49% of cases (n = 18), and there was an ANC nadir of 0 in 27% (n = 10).

At the time of neutropenia, 40% (12/30) were receiving concurrent antibiotics (4 daptomycin, 3 rifampin, 2 clindamycin, 1 rifampin plus trimethoprim-sulfamethoxazole, 1 rifampin and linezolid, and 1 both oral metronidazole and vancomycin). Concurrent renal insufficiency (CrCl < 60 mL/min) was noted among 26% (7/27) who developed neutropenia while receiving ceftaroline.

Additional symptoms at the time of neutropenia were reported: 6 (16%) had fevers, 3 (8%) abdominal pain, 2 (5%) rash, 1 (3%) nausea/vomiting, and 1 (3%) bleeding. Complications of neutropenia included a single case of *Enterobacter* bacteremia. Eleven (30%) cases received G-CSF (range of 2–8 doses) for management of neutropenia. All cases survived and had a favorable outcome.

Four studies (including the current study) included both cases developing neutropenia associated with ceftaroline use and those without neutropenia. From these studies, incidence and identified associated factors (excluding the current study) are summarized below.

The first study [17] was conducted at a medical center in Baton Rouge, Louisiana (2012–2014), and reported 39 patients who received ≥7 days of ceftaroline, of whom 7 (18%) developed neutropenia after a median duration of therapy (range) of 27 (9–125) days. The authors did not find an association in univariate analyses between ceftaroline duration or dosage and the development of neutropenia. In their analyses, female sex and lower body mass index (BMI) were associated with neutropenia.

A second study was conducted at 2 hospitals in Boston examining ≥7 days of ceftaroline use (2010–2015), in which a total of 67 patients were studied and 5 (7%) developed neutropenia [16]. Of note, 2 cases included in their original report were excluded in our analyses as the ANC nadir was not <1500 cells/mm^3^. Those who developed neutropenia had a longer median (range) duration of therapy (25 [13–68] days) compared with those not developing neutropenia (15 [ 7–64] days); however, no associations with other factors including demographics, underlying conditions, BMI, or concurrent antibiotic use were demonstrated.

A third study examined patients receiving ≥14 days of ceftaroline compared with those receiving other antibiotics for *S. aureus* infections (eg, cefazolin, daptomycin, linezolid, nafcillin, or vancomycin) [28]. This study found that 17% (9/53) of the ceftaroline group developed neutropenia (ie, ANC < 1500 cells/mm^3^), an incidence 4-fold higher than the comparator group. Younger age and bone or joint infections were risk factors for neutropenia. Duration of ceftaroline use was not significantly different between those with and without neutropenia, and although females were more likely to develop neutropenia, this did not reach significance (personal communication with R. Brigg Turner, PharmD, School of Pharmacy, Pacific University, May 14, 2018).

The combined incidence of neutropenia in these 4 studies that evaluated ceftaroline use for ≥7–14 days was 11.6% (25/215 total patients; range, 7%–18%). An important consideration when interpreting these results is that these studies occurred at separate institutions with different patient populations and study designs; thus there is a notable limitation to combining these data. Overall, factors associated with the development of neutropenia were inconsistent among the evaluable studies.

## DISCUSSION

Neutropenia is an increasingly recognized complication associated with the long-term use of ceftaroline. Ceftaroline utilized for short durations (5–14 days) for its FDA-approved indications of ABSSSI and CABP is associated with minimal risk of neutropenia. However, as ceftaroline is increasingly utilized for durations and doses greater than those studied in the initial clinical trials [10], safety data in these settings are needed. Our case series and review of the literature suggest that extended-duration ceftaroline use (ie, ≥14 days) is associated with a substantial risk of incident neutropenia of approximately 12%, which appears higher than for other β-lactam antibiotics. In our review, the median time from ceftaroline initiation to neutropenia (range) was 25 (8–125) days, with only 2 cases with a duration of ≤14 days of use. It is unlikely that neutropenia would have an onset before 7 days, based on the lack of neutropenia in phase III trials, where the approximate mean/median duration was 7 days [5–8]. The reason for variance in the percentage of ceftaroline-associated neutropenia (range, 7%–18%) between studies is unknown but is likely related to differences in study populations or an unrecognized risk factor.

Although studies have examined risk factors and their association with neutropenia during ceftaroline use, data thus far remain inconclusive. Single studies have suggested antibiotic duration [16], female sex [17], low BMI [17], younger age [28], and bone and joint infections [28] to be significant factors in neutropenia development; however, these factors have not been replicated by other studies. Interestingly, almost half of our cases were being treated for bone and/or joint infections, and previous studies have observed neutropenia in patients being treated with antimicrobials for bone-related infections [30, 31]. This may be related to extended duration of treatment for these types of infection (eg, often for 6 weeks). There are other possible risk factors including the daily dose of ceftaroline, particularly in those with renal insufficiency, as ceftaroline’s primary route of elimination is renal (88%) [21]. Both the half-life and area under the concentration-time curve (AUC) increase with renal impairment compared with normal renal function (CrCl > 80 mL/min) by 1.27 and 1.58 times for half-life, and 1.19 and 1.52 times for AUC with mild (CrCl > 50–80 mL/min) and moderate (CrCl > 30–50 mL/min) renal impairment, respectively [32]. Additionally, concomitant medications and pretreatment exposure to other agents (including prior antibiotics) with residual bone marrow effects may be potential risk factors [33, 34]. Forty percent (12/30) of cases were on concurrent antibiotics, with 67% (8/12) of these receiving antibiotics associated with neutropenia, notably trimethoprim-sulfamethoxazole, clindamycin, rifampin, linezolid, metronidazole, and vancomycin [33, 34]. However, the role of these prior drugs/antibiotics in the development of neutropenia remains unclear as these factors were not investigated in prior ceftaroline case:control data analyses [16, 17, 28]. As ceftaroline is often used as salvage therapy, other patient and disease factors may play a role; however, at least 1 study has questioned this by examining similar agents used for salvage therapy [17].

Although all β-lactams may be associated with neutropenia, the risk of ceftaroline appears higher than reported in the literature for other cephalosporins. It is important to recognize that the reported incidence varies in the literature based on study population and study design. For cefazolin, several studies have suggested that the incidence of neutropenia is 2%–4% in those receiving longer than 2 weeks of therapy [28, 35]. Other comparative drugs (vancomycin, daptomycin, nafcillin, linezolid) had substantially lower incidence of neutropenia [28].

The mechanism whereby ceftaroline (or other cephalosporins) leads to neutropenia remains unclear; however, both immune-mediated and bone marrow effects (via immune-mediated processes and/or direct damage to the myeloid cell line) have been proposed [36–38]. Of note, some cases of ceftaroline-associated neutropenia also had notable reductions in red blood cell and platelet counts, although this was not universal, suggesting a possible diffuse bone marrow effect. Some cases also had fever, rash, and/or eosinophilia, suggesting a potential immune or IgE-mediated phenomena [13].

Clinical applications of these data include the need for regular complete blood counts (CBCs) while receiving ceftaroline. The rapid rate of ANC decline in many of the cases is notable (Figure 1A–D); hence close follow-up is needed. We recommend that CBCs be obtained at baseline and at least weekly. If a decline in the ANC is detected, then biweekly or more frequent monitoring should be instituted. The current study also advocates for clinician awareness regarding the risk of neutropenia, which exceeds that stated in the package insert for ceftaroline (<2%) [21], particularly when utilized for durations ≥14 days.

The clinical management of ceftaroline-associated neutropenia includes rapid antibiotic discontinuation after ruling out other causes, especially if the ANC is <1500 cells/mm^3^ [33]. However, even earlier discontinuation should be considered. It is notable that 27% developed an ANC nadir of 0 cells/mm^3^. G-CSF was utilized in 30% of the cases in the literature to assist in neutrophil recovery. Even though there are limited trials evaluating the use of G-CSF for drug-induced neutropenia, G-CSF may be considered in high-risk patients including those with ANCs of less than 100 cells/mm^3^ due to increased risk of mortality in these patients [33, 39]. Various reports demonstrate a reduction in duration of neutropenia, antibiotic therapy, and length of hospital stay with the use of G-CSF [33, 39]. Although neutropenic complications in cases in this review were uncommon and all patients had favorable outcomes, clinical vigilance is advised, especially in the setting of severe infections (eg, bacteremia, endocarditis).

This review has several strengths, including being the most comprehensive review on ceftaroline-associated neutropenia to date. In addition, as several of the reports lacked detailed and/or individualized data, this review included additional data obtained after contacting authors of prior reports. The limitations of our study include the overall small number of patients in the published literature who have received extended durations of ceftaroline in which neutropenia was described. Additionally, data for the literature review originated from multiple studies with diverse demographic distributions, patient comorbidities, and study designs, which is important to consider when interpreting the combined findings. As the practice of using ceftaroline for ≥14 days is increasingly common, more studies are needed to more precisely define the incidence of neutropenia in this setting and to identify risk factors for its occurrence. Furthermore, there was notable heterogeneity among published reports in the data available, including complete host comorbidities, definitions of neutropenia utilized, and the timing (as the first agent vs as a salvage regimen) and duration of ceftaroline use. Finally, although scoring systems (eg, Naranjo), temporal associations, and the resolution of neutropenia after drug discontinuation support the associations between ceftaroline and the development of neutropenia, a causative relationship cannot be proven without a prospective trial.

In summary, ceftaroline-associated neutropenia is an overall common phenomenon when utilized for ≥14 days. Thrombocytopenia, anemia, and eosinophilia are also possible. Complete blood count monitoring is recommended during ceftaroline use, and twice-weekly monitoring should be considered with duration ≥14 days and/or when an ANC reduction is detected. A reduction in ANC should prompt rapid drug discontinuation as agranulocytosis may rapidly occur. Postmarketing data on possible adverse events associated with novel antibiotics are critically important, especially when utilized off-label for extended durations. Further research is needed to determine the mechanism and risk factors for the high incidence of neutropenia associated with long-term ceftaroline use.

## Supplementary Data

Supplementary materials are available at *Open Forum Infectious Diseases* online. Consisting of data provided by the authors to benefit the reader, the posted materials are not copyedited and are the sole responsibility of the authors, so questions or comments should be addressed to the corresponding author.

ofz168_suppl_supplementary_table_1

## Notes

Prior presentation. This information was presented as poster #2426 at IDWeek 2018; October 6, 2018; San Francisco, CA.

## References

[1] Naber CK *Staphylococcus aureus* bacteremia: epidemiology, pathophysiology, and management strategies. Clin Infect Dis 2009; 48(Suppl 4):S231–7. 19374578 10.1086/598189

[2] Bergin SP, Holland TL, Fowler VG Jr, Tong SYC Bacteremia, sepsis, and infective endocarditis associated with *Staphylococcus aureus*. Curr Top Microbiol Immunol 2017; 409:263–96. 26659121 10.1007/82_2015_5001

[3] Britt RS, Evoy KE, Lee GC, et al. Early use of ceftaroline fosamil in the United States Veterans Health Care System. Drugs 2017; 77:1345–51. 28664412 10.1007/s40265-017-0785-2 PMC5553123

[4] Meaney CJ, Hynicka LM, Tsoukleris MG Vancomycin-associated nephrotoxicity in adult medicine patients: incidence, outcomes, and risk factors. Pharmacotherapy 2014; 34:653–61. 24700598 10.1002/phar.1423

[5] File TM Jr, Low DE, Eckburg PB, et al. FOCUS 1: a randomized, double-blinded, multicentre, phase III trial of the efficacy and safety of ceftaroline fosamil versus ceftriaxone in community-acquired pneumonia. J Antimicrob Chemother 2011; 66(Suppl 3):iii19–32. 21482566 10.1093/jac/dkr096

[6] Low DE, File TM Jr, Eckburg PB, et al. FOCUS 2: a randomized, double-blinded, multicentre, phase III trial of the efficacy and safety of ceftaroline fosamil versus ceftriaxone in community-acquired pneumonia. J Antimicrob Chemother 2011; 66(Suppl 3):iii33–44. 21482568 10.1093/jac/dkr097

[7] Corey GR, Wilcox MH, Talbot GH, et al. CANVAS 1: the first phase III, randomized, double-blind study evaluating ceftaroline fosamil for the treatment of patients with complicated skin and skin structure infections. J Antimicrob Chemother 2010; 65(Suppl 4):iv41–51. 21115454 10.1093/jac/dkq254

[8] Wilcox MH, Corey GR, Talbot GH, et al. CANVAS 2: the second phase III, randomized, double-blind study evaluating ceftaroline fosamil for the treatment of patients with complicated skin and skin structure infections. J Antimicrob Chemother 2010; 65(Suppl 4):iv53–iv65. 21115455 10.1093/jac/dkq255

[9] Allergan. Allergan Announces FDA Approval of updated label for TEFLARO® (ceftaroline fosamil). https://www.allergan.com/news/news/thomson-reuters/allergan-announces-fda-approval-of-updated-lab-(1). Accessed 1 May 2018.

[10] Cosimi RA, Beik N, Kubiak DW, Johnson JA Ceftaroline for severe methicillin-resistant *Staphylococcus aureus* infections: a systematic review. Open Forum Infect Dis 2017; 4(X):XXX–XX. 10.1093/ofid/ofx084 PMC5499876 28702467

[11] Vidaillac C, Leonard SN, Rybak MJ In vitro activity of ceftaroline against methicillin-resistant *Staphylococcus aureus* and heterogeneous vancomycin-intermediate *S. aureus* in a hollow fiber model. Antimicrob Agents Chemother 2009; 53:4712–7. 19738009 10.1128/AAC.00636-09 PMC2772315

[12] Matzneller P, Lackner E, Lagler H, et al. Single- and repeated-dose pharmacokinetics of ceftaroline in plasma and soft tissues of healthy volunteers for two different dosing regimens of ceftaroline fosamil. Antimicrob Agents Chemother 2016; 60:3617–25. 27044549 10.1128/AAC.00097-16 PMC4879389

[13] Olaison L, Belin L, Hogevik H, Alestig K Incidence of beta-lactam-induced delayed hypersensitivity and neutropenia during treatment of infective endocarditis. Arch Intern Med 1999; 159:607–15. 10090118 10.1001/archinte.159.6.607

[14] Jain R, Chan JD, Rogers L, et al. High incidence of discontinuations due to adverse events in patients treated with ceftaroline. Pharmacotherapy 2014; 34:758–63. 24807197 10.1002/phar.1435

[15] Varada NL, Sakoulas G, Lei LR, Chua J Agranulocytosis with ceftaroline high-dose monotherapy or combination therapy with clindamycin. Pharmacotherapy 2015; 35:608–12. 26037689 10.1002/phar.1596

[16] Furtek KJ, Kubiak DW, Barra M, et al. High incidence of neutropenia in patients with prolonged ceftaroline exposure. J Antimicrob Chemother 2016; 71:2010–3. 27076105 10.1093/jac/dkw062 PMC4896407

[17] LaVie KW, Anderson SW, O’Neal HR Jr, et al. Neutropenia associated with long-term ceftaroline use. Antimicrob Agents Chemother 2016; 60:264–9. 26503657 10.1128/AAC.01471-15 PMC4704231

[18] Dellabella A, Roshdy D, Martin KE High incidence of adverse effects with extended use of ceftaroline. Ann Pharmacother 2016; 50:1068–9. 27586432 10.1177/1060028016667583

[19] Naranjo CA, Busto U, Sellers EM, et al. A method for estimating the probability of adverse drug reactions. Clin Pharmacol Ther 1981; 30:239–45. 7249508 10.1038/clpt.1981.154

[20] Blumenthal KG, Kuhlen JL Jr, Weil AA, et al. Adverse drug reactions associated with ceftaroline use: a 2-center retrospective cohort. J Allergy Clin Immunol Pract 2016; 4:740–6. 27130709 10.1016/j.jaip.2016.03.008 PMC4939098

[21] TEFLARO (ceftaroline fosamil) package insert. https://www.allergan.com/assets/pdf/teflaro_pi. Accessed 23 March 2018.

[22] Majumdar R, Crum-Cianflone NF Telavancin for MRSA endocarditis: case report and review of the literature. Infect Dis Clin Pract 2017; 25:176–183.

[23] Rimawi RH, Frenkel A, Cook PP Ceftaroline - a cause for neutropenia. J Clin Pharm Ther 2013; 38:330–2. 23590618 10.1111/jcpt.12062

[24] Yam FK, Kwan BK A case of profound neutropenia and agranulocytosis associated with off-label use of ceftaroline. Am J Health Syst Pharm 2014; 71:1457–61. 25147169 10.2146/ajhp130474

[25] Sahar N, Ratajczak T, Conger NG Ceftaroline-induced agranulocytosis. J Med Cases 2016; 7:197–201.

[26] Phull P, Lerner A Agranulocytosis secondary to ceftaroline use: a case report and review of the literature. J Hematol 2016; 5:103–105.

[27] Khan U, Hadid T Fever, rash and agranulocytosis. BMJ Case Rep. ** 2017; doi:10.1136/bcr-2017-219403**. 10.1136/bcr-2017-219403 PMC5353480 28270401

[28] Turner RB, Wilson DE, Saedi-Kwon H, et al. Comparative analysis of neutropenia in patients receiving prolonged treatment with ceftaroline. J Antimicrob Chemother 2018; 73:772–8. 29237024 10.1093/jac/dkx452

[29] Zasowski EJ, Trinh TD, Claeys KC, et al. Multicenter observational study of ceftaroline fosamil for methicillin-resistant *Staphylococcus aureus* bloodstream infections. Antimicrob Agents Chemother. 2017; doi: 10.1128/AAC.02015-16. 10.1128/AAC.02015-16 PMC5278749 27895012

[30] McCluskey WP, Esterhai JL Jr, Brighton CT, Heppenstall RB Neutropenia complicating parenteral antibiotic treatment of infected nonunion of the tibia. Arch Surg 1989; 124:1309–12. 2818186 10.1001/archsurg.1989.01410110067013

[31] Peralta FG, Sánchez MB, Roíz MP, et al. Incidence of neutropenia during treatment of bone-related infections with piperacillin-tazobactam. Clin Infect Dis 2003; 37:1568–72. 14614681 10.1086/379519

[32] Steed ME, Rybak MJ Ceftaroline: a new cephalosporin with activity against resistant gram-positive pathogens. Pharmacotherapy 2010; 30:375–89. 20334458 10.1592/phco.30.4.375

[33] Andrès E, Mourot-Cottet R Non-chemotherapy drug-induced neutropenia - an update. Expert Opin Drug Saf 2017; 16:1235–42. 28879784 10.1080/14740338.2017.1376645

[34] Curtis BR Non-chemotherapy drug-induced neutropenia: key points to manage the challenges. Hematology Am Soc Hematol Educ Program 2017; 2017:187–93. 29222255 10.1182/asheducation-2017.1.187 PMC6142577

[35] Youngster I, Shenoy ES, Hooper DC, Nelson SB Comparative evaluation of the tolerability of cefazolin and nafcillin for treatment of methicillin-susceptible *Staphylococcus aureus* infections in the outpatient setting. Clin Infect Dis 2014; 59:369–75. 24785233 10.1093/cid/ciu301 PMC4110443

[36] Murphy MF, Minchinton RM, Metcalfe P, et al. Neutropenia due to beta lactamine antibodies. Br Med J 1984; 288:795. 10.1136/bmj.288.6419.795 PMC1444666 6423084

[37] Deldar A, Lewis H, Bloom J, Weiss L Cephalosporin-induced changes in the ultrastructure of canine bone marrow. Vet Pathol 1988; 25:211–8. 3394212 10.1177/030098588802500305

[38] Neftel KA, Hübscher U Effects of beta-lactam antibiotics on proliferating eucaryotic cells. Antimicrob Agents Chemother 1987; 31:1657–61. 3324959 10.1128/aac.31.11.1657 PMC175015

[39] Andrès E, Maloisel F, Zimmer J The role of haematopoietic growth factors granulocyte colony-stimulating factor and granulocyte-macrophage colony-stimulating factor in the management of drug-induced agranulocytosis. Br J Haematol 2010; 150:3–8. 20151980 10.1111/j.1365-2141.2010.08104.x

